# The causal effects of immune cells on pancreatic cancer: A 2‑sample Mendelian randomization study

**DOI:** 10.1097/MD.0000000000037797

**Published:** 2024-04-19

**Authors:** Xinyun Zou, Jinlan Shen, Xiaomei Yong, Yong Diao, Ling Zhang

**Affiliations:** a Department of Oncology, People’s Liberation Army The General Hospital of Western Theater Command, Chengdu, China; b Department of Medical Laboratory, People’s Liberation Army The General Hospital of Western Theater Command, Chengdu, China.

**Keywords:** Immunology, Mendelian randomization analysis, pancreatic cancer

## Abstract

Leveraging publicly available genetic datasets, we conducted a comprehensive 2-sample Mendelian randomization (MR) analysis to explore the causal links between 731 immunophenotypes and the risk of pancreatic cancer (PC). To ensure the robustness of our findings, extensive sensitivity analyses were performed, evaluating stability, heterogeneity, and potential horizontal pleiotropy. Our analysis pinpointed 24 immunophenotypes significantly associated with the risk of PC. Notably, phenotypes such as CD4^+^ CD8dim %leukocyte (OR = 0.852, 95% CI = 0.729–0.995, *P* = .0430) and HLA DR+ CD4^+^ AC (OR = 0.933, 95% CI = 0.883–0.986) in TBNK were inversely correlated with PC risk. Conversely, phenotypes like CD28 on CD45RA− CD4 non-Treg (OR = 1.155, 95% CI = 1.028–1.297, *P* = .016) and CD25 on activated Treg (OR = 1.180, 95% CI = 1.014–1.374, *P* = .032) in Treg cells, among others, exhibited a positive correlation. These insights offer a valuable genetic perspective that could guide future clinical research in this area.

## 1. Introduction

Pancreatic cancer (PC) is increasingly recognized as one of the most malignant tumors and a leading cause of cancer mortality worldwide. In 2021, it ranked as the third leading cause of cancer-related deaths and is projected to escalate to the second position by 2030.^[1,2]^ Currently, only 20% of PC cases are diagnosed at an early, potentially resectable and curable stage. However, the insidious onset of PC, challenges in early diagnosis, and rapid progression mean that most patients lose their eligibility for surgery at the time of diagnosis. Furthermore, even among resected cases, the rate of systemic recurrence is alarmingly high, ranging from 80% to 90%.^[3]^ This situation is exacerbated by the tumor’s general insensitivity to antitumor drugs, leading to an exceedingly poor overall prognosis for patients.

PC is often characterized as “cold tumors,”^[4,5]^ exhibiting relatively few neoantigens that can be recognized by immune cells. This categorization reflects their low immunogenicity and high immunosuppressive nature, likely driven by immunodeficiency and immunosuppression within the tumor microenvironment (TME). Recent in-depth studies of the TME have shed light on the pathogenesis and potential targeted therapies for PC. Consequently, understanding the role of each immunophenotype within the PC TME is crucial for developing effective therapeutic strategies.

Mendelian randomization (MR) is an analytical method grounded in the principles of Mendel’s laws of independent assortment, primarily utilized for epidemiological etiological inference. MR leverages genetic variants associated with exposure as instrumental variables, thereby serving as proxies for the exposure itself. By observing the correlation results,^[6]^ MR facilitates the inference of potential causality, enabling a more robust assessment of the relationship between the exposure and the outcome.

In this study, we employed a comprehensive 2-sample MR analysis to elucidate the causal relationship between various immunophenotypes and PC. This approach allows for a more precise determination of how these immunophenotypes may influence the risk and progression of PC, providing valuable insights into potential therapeutic targets and strategies.

## 2. Materials and methods

### 2.1. Research design

**Figure 1.**
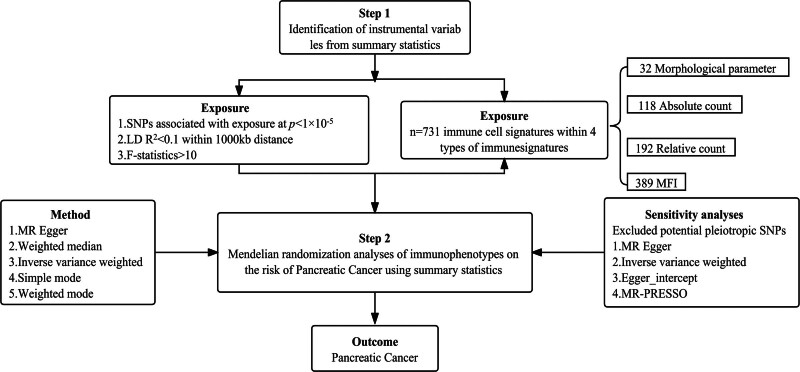
The study design of the associations of immune cells and PC. MR = Mendelian randomization, PC = Pancreatic Cancer, SNP = single nucleotide polymorphism.

The comprehensive design of our study, which aims to assess the causal association between 731 immunophenotypes and PC, is depicted in Figure 1. We utilized a 2-sample MR analysis for this purpose. MR employs genetic variations as proxies for risk factors, and the validity of instrumental variables (IVs) in causal inference hinges on 3 critical assumptions^[7]^:

*Strong association.* There must be a robust correlation between the genetic variation and the exposure factors. This ensures that the genetic variants can reliably represent the exposure.

*Independence from confounders.* The genetic variation should be independent of any confounding factors that might affect both the exposure and the outcome. This assumption is crucial to prevent bias in the causal inference.

*Exclusivity of the exposure pathway.* The genetic variation should influence the outcome exclusively through the exposure, and not via any alternative pathways. This exclusivity is vital for attributing any observed effects on the outcome directly to the exposure.

These assumptions are fundamental to the integrity and validity of the MR analysis in establishing a causal relationship between immunophenotypes and PC.

### 2.2. Genome-wide association study (GWAS) data sources for PC

The GWAS statistics utilized for PC in this study were sourced from the FinnGen research project (https://www.finngen.fi/en, R6, published in 2022). This extensive project analyzed data from 224,737 participants, encompassing a broad spectrum of diseases. Specifically, the study investigated 15 diseases, resulting in the identification of 2733 genome-wide significant associations (893 with PWS, *P* < 2.6 × 10^−11^) across 2496 (771 with PWS) independent loci and 807 (247 with PWS) endpoints.^[8]^

Among the significant findings, the FinnGen study reported 232 cases of esophageal cancer, 633 cases of gastric cancer, 1803 cases of colorectal cancer, 605 cases of PC, 304 cases of liver cancer, and included 17,406 noncancer controls. The detailed breakdown of the GWAS data sources specifically pertaining to PC, as employed in this MR study, is comprehensively presented in Table 1.

**Table 1:** Details of the genome-wide association studies and datasets used in our analyses.

Exposure or outcome	Sample size	Ancestry	Links for data download	PMID
Immunophenotypes	731 immunological characteristics	European Ancestry	http://ftp.ebi.ac.uk/pub/databases/gwas/summary_statistics/ (GCST90001391-GCST90002121)	32929287
Pancreatic cancer	287,137 controls and 1416 PC cases	FinnGen research project	https://storage.googleapis.com/finngen-public-data-r9/summary_stats/finngen_R9_C3_PANCREAS_EXALLC.gz	36653562

### 2.3. Immunity-wide GWAS data sources

The GWAS data for 731 immunological characteristics were sourced from the GWAS Catalog, encompassing serial numbers from GCST90001391 to GCST90002121.^[9]^ These immunophenotypes were categorized into 4 primary feature types: absolute cell (AC) count (*n* = 118), median fluorescence intensity (MFI) which reflects the level of surface antigen expression (*n* = 389), morphological parameter (MP) (*n* = 32), and relative cell (RC) count (*n* = 192).^[10]^

Specifically, the MFI, AC, and RC features included data on various cell types such as B cells, CDCs, T-cell maturation stages, monocytes, myeloid cells, TBNK (T-cell, B-cell, natural killer cell) cells, and Treg cells. In contrast, the MP feature was focused on CDC and TBNK cell types. The original immune trait GWAS data,^[11]^ derived from an extensive analysis of the effects of approximately 22 million variants on 731 immune cell traits in 3757 Sardinians, revealed 122 significant (*P* < 1.28 × 10^−11^) independently associated signals for 459 cellular traits across 70 loci, 53 of which were newly identified. This analysis has been instrumental in identifying several molecules and mechanisms involved in cellular regulation.

Comprehensive details of these GWAS data sources for the 731 immunological characteristics are systematically presented in Table 1. This study was a reanalysis of previously collected and published publicly available data and therefore did not require additional ethical approval.

### 2.4. Instrumental variables: selection criteria and methodology

In line with recent research findings,^[9]^ we established a significance threshold for each immunological trait’s IVs at 1 × 10^−5^. To ensure the independence of loci for our IVs, we employed the “TwoSampleMR” software package. This involved setting a linkage disequilibrium (LD) threshold at *R*^2^ < 0.001 and a distance for aggregation at 10,000 kb, referencing the 1000 Genomes Project’s European (EUR) data.

For each single nucleotide polymorphism (SNP), we meticulously extracted key information including the effect allele, the *β* value representing the effect size, the standard error, and *P* value. To evaluate the robustness of our instrumental variables, we calculated the proportion of variance explained (*R*^2^) and the *F* statistic. The formulae applied were *R*^2^ = 2 × MAF × (1 − MAF) × *β*^2^ and *F* = *R*^2^ × (*n* − *k* − 1)/[*k* × (1 − *R*^2^)], where MAF represents the minor allele frequency of the SNP, “*n*” is the sample size, and “*k*” is the number of IVs utilized.^[12,13]^

### 2.5. Methodology for statistical analysis

In our analysis, we employed a range of methods to ascertain the potential causal links between immunophenotypes and PC. These methods included fixed/random-effects inverse variance weighting (IVW), the weighted median approach, MR-Egger regression, and both simple and weighted mode techniques.

The IVW method was chosen as our primary analytical tool. This decision was based on its widespread acceptance for providing precise effect estimates in MR analyses.^[14,15]^ The IVW approach involves initially calculating individual SNP ratio estimates using the Wald estimate and the Delta method. Subsequently, these individual estimates are aggregated to derive the overall causal estimate.^[16]^

To address potential heterogeneity among the selected SNPs, Cochran’s *Q* test and MR-Egger regression were utilized. In cases where heterogeneity was detected (*P* < .05), the random-effects IVW method was applied. Conversely, in the absence of significant heterogeneity, the fixed-effects IVW method was employed.^[17]^ Given that IVW results can be influenced by the choice of instruments and possible pleiotropic effects, we conducted additional sensitivity analyses. These analyses included the weighted median method, which is particularly useful for providing more reliable causal effect estimates in scenarios where validated instruments are not readily available.^[18]^

### 2.6. Statistical tools for assessing horizontal pleiotropy and data robustness

In our study, MR-Egger regression was utilized as a key tool to evaluate horizontal pleiotropy among SNPs. A significant intercept term in the MR-Egger regression, indicated by a *P* value < .05, was considered evidence of horizontal pleiotropy.^[19]^

Additionally, to further refine our analysis, the MR-PRESSO test was employed. This test is designed to identify and exclude potential outliers among SNPs that could significantly skew our results. It achieves this through a global heterogeneity test.

To visually assess the impact of outliers and the robustness of our findings, we incorporated scatterplots and funnel plots into our analysis. Scatterplots were particularly useful in confirming that our results were not disproportionately influenced by any outliers. Meanwhile, funnel plots provided a graphical representation of the strength and consistency of the correlations, further affirming the absence of significant heterogeneity.

All 2-sample MR analyses were conducted using R version 3.6.3, available at [https://www.r-project.org/]. For these analyses, we utilized a suite of packages including “Mendelian Randomisation,” “TwoSampleMR,” “MR-PRESSO,” and “ggplot2” for data visualization and analysis.

## 3. Results

In this study, we meticulously analyzed the causal relationship between 731 immunophenotypes and PC using a multifaceted approach. Five distinct analytical methods were employed: MR-Egger, weighted median, inverse variance weighted, simple mode, and weighted mode. This comprehensive analysis led to the identification of 24 immunophenotypes significantly correlated with PC, as detailed in Table S1, Supplemental Digital Content, http://links.lww.com/MD/M167.

To rigorously assess the horizontal pleiotropy, heterogeneity, and sensitivity of each immunophenotype (referenced in Table S1, Supplemental Digital Content, http://links.lww.com/MD/M167), we employed 3 robust validation methods: IVW, the MR-Egger method, and the MR-PRESSO method. This validation process was critical in ensuring the reliability of our findings. We meticulously combined data from these MR analyses, confirming the concordance between exposure and outcome variables (Table S2, Supplemental Digital Content, http://links.lww.com/MD/M168), and also provided detailed outcome data for each SNP in Table S3, Supplemental Digital Content, http://links.lww.com/MD/M169.

**Figure 2.**
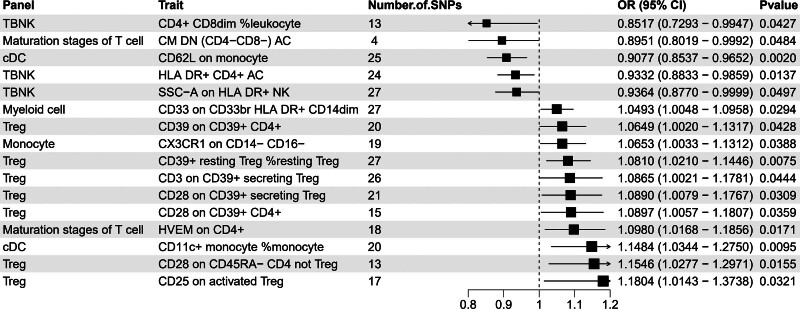
Forest plots showed the causal associations between PC and immunophenotypes by using IVW. CI = confidence interval, IVW = inverse variance weighting, PC = Pancreatic Cancer.

Our results, based on the odds ratios (ORs) and *P* values derived from the MR analysis, highlighted the most significant correlations (Fig. 2). Notably, in the TBNK cell subset, CD4^+^ CD8dim %leukocyte (OR = 0.852, 95% CI = 0.729–0.995, *P* = .0430), HLA DR+ CD4^+^ AC (OR = 0.933, 95% CI = 0.883–0.986, *P* = .014), and SSC-A on HLA DR+ NK cells (OR = 0.936, 95% CI = 0.877–1.000, *P* = .049) were found to inhibit PC. Similarly, in mature T cells, CM DN (CD4^−^ CD8^−^) AC (OR = 0.895, 95% CI = 0.802–0.999, *P* = .048), and CD62L on monocytes (OR = 0.908, 95% CI = 0.854–0.965, *P* = .002) in cDCs were also identified as inhibitors of PC.

Furthermore, our results revealed several immunophenotypes positively correlated with PC, indicating a potential promotional effect on PC. Notably, within the Treg cell subset, CD39 on CD39+ CD4^+^ (OR = 1.065, 95% CI = 1.002–1.132, *P* = .043), CD39+ resting Treg %resting Treg (OR = 1.081, 95% CI = 1.021–1.145, *P* = .007), CD3 on CD39+ secreting Treg (OR = 1.087, 95% CI = 1.002–1.178, *P* = .0444), CD28 on CD39+ secreting Treg (OR = 1.089, 95% CI = 1.008–1.177, *P* = .031), CD28 on CD39+ CD4^+^ (OR = 1.090, 95% CI = 1.006–1.181, *P* = .036), CD28 on CD45RA− CD4 non-Treg (OR = 1.155, 95% CI = 1.028–1.297, *P* = .016), and CD25 on activated Treg (OR = 1.180, 95% CI = 1.014–1.374, *P* = .032) were identified as having a significant positive correlation with PC risk.

In the myeloid cell subset, CD33 on CD33br HLA DR+ CD14dim (OR = 1.049, 95% CI = 1.005–1.096, *P* = .029) and Monocyte CX3CR1 on CD14^−^ CD16^−^ (OR = 1.065, 95% CI = 1.003–1.131, *P* = .039), as well as HVEM on CD4^+^ (OR = 1.098, 95% CI = 1.017–1.186, *P* = .017) in mature T cells and CD11c+ monocyte %monocyte (OR = 1.148, 95% CI = 1.034–1.275, *P* = .009) in cDCs, were also positively correlated with an increased risk of PC.

The robustness of these observed causal relationships was further validated by the results of the other 4 MR analysis methods and sensitivity analyses. The MR-Egger’s intercept and MR-PRESSO’s global test effectively ruled out the possibility of horizontal pleiotropy, as detailed in Table S1, Supplemental Digital Content.

## 4. Discussion

**Figure 3.**
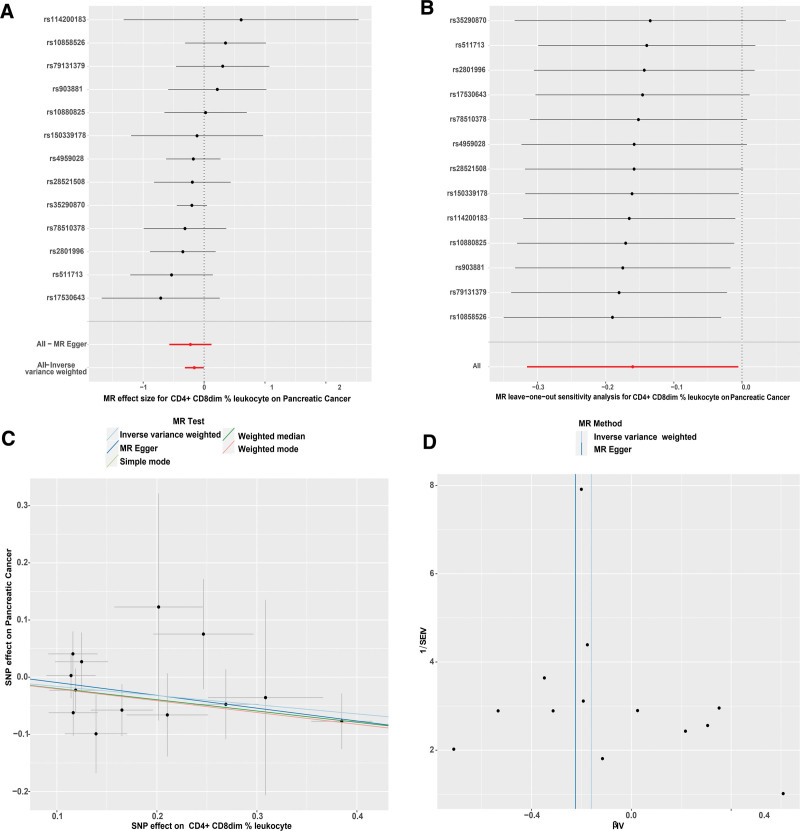
(A) Forest plot of the causal effects of single nucleotide polymorphisms associated with immune cells on PC. (B) Leave-one-out of SNPs associated with CD4^+^ CD8dim %leukocyte and their risk of PC. Each black point represents result of the IVW MR method applied to estimate the causal effect of CD4^+^ CD8dim %leukocyte on PC excluding particular SNP. (C) Scatter plots of genetic associations with CD4^+^ CD8dim %leukocyte against the genetic associations with PC. (D) Funnel plot to assess heterogeneity. PC = Pancreatic Cancer, SNP = single nucleotide polymorphism.

Our analysis highlighted that certain immunophenotypes exhibited an inhibitory effect on PC. Notably, CD4^+^ CD8dim %leukocyte emerged as the immunophenotype with the most pronounced negative correlation to PC risk. The MR findings for this immunophenotype indicated a high degree of stability and absence of horizontal pleiotropy and heterogeneity, as illustrated in Figure 3.

NK cells, constituting 5% to 20% of human circulating lymphocytes^[20,21]^, play a pivotal role in innate immunity. As nonspecific cytotoxic cells, NK cells can recognize and eliminate virus-infected or tumor cells without prior antigenic stimulation, primarily through cytotoxic receptors like CD16. Their significance in tumor growth control is well-documented in experimental mouse models, underscoring their potential in cancer immunotherapy.^[22,23]^

In the context of adaptive immunity, CD4^+^ T-cells in NK cells are known to recognize antigens restricted by Major Histocompatibility Complex (MHC) II molecules, while CD8+ T-cells target antigens presented by MHC I molecules on the surfaces of infected or malignant cells. This interaction is crucial for initiating specific immune responses.^[24]^

Additionally, HLA DR, a human MHC II antigen, is expressed on B lymphocytes, monocytes, and macrophages. It plays a vital role in antigen presentation to CD4^+^ T-cells, thereby facilitating T-cell proliferation and stimulating B-cell-mediated humoral immunity.^[25]^ The relationship between NK cell dynamics and PC is particularly noteworthy. Lee et al^[26]^ observed a decline in NK cell activity in PC patients correlating with disease progression and poorer clinical outcomes. This finding suggests an inhibitory role of NK cells in the context of PC, highlighting their potential as a therapeutic target.

Recent findings indicate that CD62L expression on monocytes within dendritic cells and CM DN (CD4^−^ CD8^−^) AC in mature T-cells are associated with a reduced risk of PC. Analysis of PC tissues has revealed a diminished presence of dendritic cells, characterized by lower expression of co-stimulatory molecules and cell maturation markers. This reduction compromises their antigen-presenting capacity, leading to an impaired T-cell response against tumor cells.

Furthermore, studies have shown that in the absence of dendritic cells, CD4^+^ T cells in the PC microenvironment tend to differentiate into Th17 (helper T-cell 17) cells. These cells secrete pro-inflammatory IL-17 (interleukin-17), contributing to an environment conducive to tumor growth and metastasis. Conversely, augmenting dendritic cell numbers in PC could significantly enhance the immune response against the tumor. This strategy could be synergistically combined with immunotherapeutic treatments, such as immune checkpoint inhibitors, to improve their efficacy in combating PC, often referred to as the “king of cancers” due to its aggressive nature.^[27]^

This study highlights the potential of neoantigen-specific T cells in PC treatment. In a mouse model of PC, treatment with a combination of PD-1 and PD-L1 inhibitors led to the persistence of these T cells, which expressed the central memory marker CD62L. This expression is indicative of the formation of an immune memory, significantly inhibiting tumor growth and prolonging survival in mice.^[28]^ Further research by Zhou et al^[29]^ explored the cytotoxic mechanisms of CD4 and CD8 double-negative T-cells against PC cells. Utilizing a PC xenograft model in nude mice and employing assays such as CCK-8, human recombinant protein blocking, TUNEL, and immunohistochemistry, their findings revealed that these double-negative T-cells exerted a cytotoxic effect on PC cells, inhibiting their growth both in vitro and in vivo.

However, the response to immunotherapy in PC remains complex. Despite the presence of neoantigens and activatable T cells in some patients, there is often a lack of response to immunotherapeutic approaches.^[30,31]^ PC is characterized as a “cold tumor,” marked by a paucity of CD8+ T-cells within the tumor microenvironment, as observed in both human samples and mouse models.^[4,5]^ Our MR study corroborates this observation, showing that out of 24 immune cell phenotypes with a causal link, 16 were positively associated with PC, suggesting an immune evasion profile characteristic of this malignancy.

**Figure 4.**
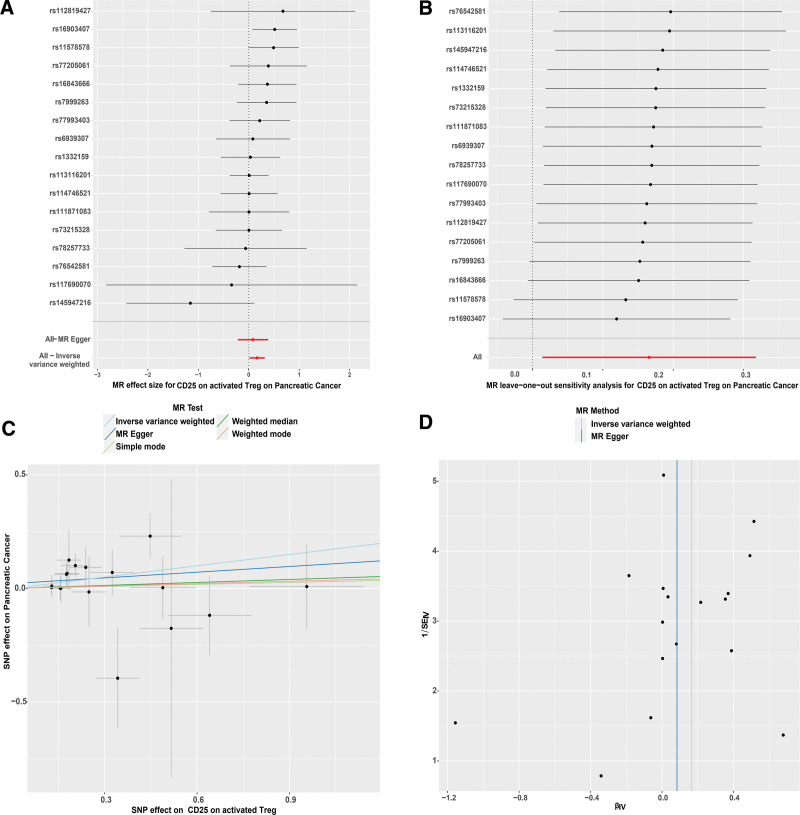
(A) Forest plot of the causal effects of single nucleotide polymorphisms associated with immune cells on PC. (B) Leave-one-out of SNPs associated with CD25 on activated Treg and their risk of PC. Each black point represents result of the IVW MR method applied to estimate the causal effect of CD25 on activated Treg on PC excluding particular SNP. (C) Scatter plots of genetic associations with CD25 on activated Treg against the genetic associations with PC. (D) Funnel plot to assess heterogeneity. PC = Pancreatic Cancer, SNP = single nucleotide polymorphism.

Our MR analysis revealed that among the 16 immunophenotypes positively influencing PC, 7 belong to the regulatory T-cell (Treg) subset. This finding underscores the significant role of Tregs in the progression of PC. Notably, the phenotype CD25 on activated Treg demonstrated the strongest positive correlation with PC. The robustness of this association was confirmed by the stability of the MR results, which showed no evidence of horizontal pleiotropy or heterogeneity (Fig. 4).

Previous studies have indicated an increased prevalence of Tregs both in the peripheral blood and tumor microenvironment of PC patients. This increase is observable not only in advanced stages of the disease but also in early precancerous lesions, suggesting a contributory role of Tregs in the disease’s progression.^[32,33]^ Our analysis further revealed that 6 of the 7 Treg immunophenotypes are characterized by the presence of CD28 and CD39 molecules, highlighting their potential significance in Treg function.

It has been established that the downregulation of CD28, a co-stimulatory molecule, is indicative of T-cell senescence. An increase in CD28-negative Tregs has been observed in various solid and hematological tumors. This phenomenon suggests that tumor-induced T-cell senescence could be an alternative mechanism through which tumors develop immune resistance.^[34]^

CD39, or nucleoside triphosphate diphosphohydrolase-1 (NTPDase1), plays a pivotal role in modulating the tumor immune response.^[35]^ CD39 facilitates the proliferation of Tregs by activating their receptors and enhances immunosuppression through increased expression of immunosuppressive receptors.^[36]^ In the context of PC, CD39 is highly expressed in cancer-associated fibroblasts (CAFs)^[37]^. Studies using a mouse model of chronic pancreatitis and fibrosis revealed that CD39 deficiency led to a marked reduction in fibrosis and elevated levels of antifibrotic IFN-γ^[38]^, suggesting a role for CD39 in promoting pancreatic tissue fibrosis.^[37]^

Our MR analysis identified CD25 on activated Treg cells as having the strongest positive correlation with PC risk. CD25, the alpha subunit of the interleukin-2 receptor (IL-2Rα), is predominantly expressed in Treg cells. It is a critical marker for Tregs and activates signaling pathways such as JAK/STAT5, PI3K/Akt, and MAPK, which are integral to cell growth, survival, differentiation, and immunity. Flow cytometry analysis of CD4 CD25 T cells as a percentage of total CD4 cells in PC patients suggests that increased Treg levels may contribute to immunosuppression and tumor progression.^[39]^ Additionally, therapeutic strategies targeting CD25 in Treg cells^[40–42]^ have shown promise in the treatment of PC, highlighting the potential of anti-CD25 immunotherapy in this context.

This MR analysis revealed that specific immunophenotypes within myeloid cells, monocytes, mature T cells, and conventional dendritic cells (cDCs) contribute to the progression of PC. These include CD33 on CD33br HLA DR+ CD14dim myeloid cells, CX3CR1 on CD14^−^ CD16^−^ monocytes, HVEM on CD4^+^ mature T-cells, and CD11c+ monocyte percentage in cDCs.

Myeloid cells are integral to the initiation of inflammation,^[43]^ a known driver of malignancies, including PC.^[44]^ Notably, HVEM is moderately expressed in the pancreas.^[45]^ CX3CR1, a chemokine receptor on microglia and macrophages in the CNS, is activated by neuron-secreted CX3CL1. High CX3CR1 expression and perineuronal infiltration are associated with local and early tumor recurrence. This suggests a role for the CX3CR1 receptor in the neurophilic mechanism of PC and its potential as an independent risk factor for early local tumor recurrence postresection.^[46]^

This study utilized published GWAS data to conduct a 2-sample MR analysis, leveraging a large sample size and high statistical power. The findings are grounded in genetic instrumental variables, with causal inferences drawn from multiple MR methodologies. The robustness of the results is underscored by their resistance to confounding factors, including horizontal pleiotropy. However, it is important to acknowledge the study’s limitation due to its reliance on a Finnish database. This raises questions about the applicability of these findings to our understanding of the immune cell profile in PC, warranting further research and data validation across diverse populations.

## 5. Conclusions

Our extensive 2-sample MR analysis has elucidated a causal link between 24 distinct immune cell phenotypes and PC. This revelation underscores the intricate interplay between the immune system and PC, opening new pathways for exploring the underlying biological mechanisms and potential immunotherapeutic strategies for this malignancy.

## Acknowledgments

We thank the FinnGen research project for developing and curating their data resources of GWAS and we also thank Orrù et al for providing immunity-wide GWAS data sources for our analyses.

## Author contributions

Data curation: Xinyun Zou, Yong Diao.

Formal analysis: Xinyun Zou, Yong Diao.

Methodology: Xinyun Zou, Jinlan Shen.

Software: Xinyun Zou.

Visualization: Xinyun Zou.

Writing—original draft: Xinyun Zou.

Investigation: Jinlan Shen, Xiaomei Yong.

Project administration: Jinlan Shen, Ling Zhang.

Validation: Xiaomei Yong, Ling Zhang.

Supervision: Ling Zhang.

## Supplementary Material






