# Supplementary material for: The causal effects of immune cells on pancreatic cancer: A 2‑sample Mendelian randomization study
Source: Medicine (Baltimore). 2024 Apr 19;103(16):e37797. doi: 10.1097/MD.0000000000037797 (PMC11029941; doi:10.1097/MD.0000000000037797)
Supplement: Supplementary file 1 [file medi-103-e37797-s001.pdf]

Online Supplementary Material

Xin Yun Zou,et al., Association Between Immune Cells And pancreatic cancer：a two-sample Mendelian randomization analysis

Table S1 Exploration of The Causal Effect of Immunophenotypes Onset On PC in MR analyses

Abbreviations: CI, confidence interval; PC, pancreatic cancer; MR, Mendelian randomization; OR, odds ratio; SNP, single nucleotide polymorphism.

Supplementary Table 1 Exploration of The Causal Effect of Immunophenotypes Onset On PC in MR analyses

Immunophenotypes	*F* mean	Methods	N.SNP	OR	95% CI	OR/*P*val	Pleiotropy/Pval	heterogeneity/Pval
Absolute count								
CD4 Treg AC								
	21.97	MR Egger	21	0.98	0.85-1.15	0.812	0.125	0.499
		Weighted median	21	0.95	0.82-1.10	0.483		
		Inverse variance weighted	21	0.89	0.81-0.98	0.020		0.402
		Simple mode	21	0.93	0.74-1.15	0.497		
		Weighted mode	21	0.94	0.82-1.08	0.407		
CM DN (CD4-CD8-) AC	21.33							
		MR Egger	4	0.90	0.74-1.11	0.438	0.916	0.169
		Weighted median	4	0.90	0.81-1.01	0.082		
		Inverse variance weighted	4	0.90	0.80-1.00	0.048		0.311
		Simple mode	4	0.94	0.79-1.10	0.483		
		Weighted mode	4	0.91	0.81-1.02	0.195		
HLA DR+ CD4+ AC								
	25.52	MR Egger	24	0.91	0.85-0.98	0.017	0.309	0.875
		Weighted median	24	0.93	0.86-1.00	0.078		
		Inverse variance weighted	24	0.93	0.88-0.99	0.014		0.864
		Simple mode	24	0.94	0.82-1.01	0.443		
		Weighted mode	24	0.93	0.87-1.00	0.050		
HLA DR+ CD8br AC								
	29.38	MR Egger	34	1.03	0.95-1.11	0.495	0.255	0.772
		Weighted median	34	1.03	0.95-1.12	0.434		
		Inverse variance weighted	34	1.06	1.00-1.12	0.037		0.753
		Simple mode	34	1.05	0.90-1.23	0.531		

		Weighted mode	19	1.05	0.97-1.23	0.278		
CD8dim NKT AC								
	28.99	MR Egger	26	1.09	0.97-1.22	0.146	0.921	0.588
		Weighted median	26	1.09	0.96-1.24	0.178		
		Inverse variance weighted	26	1.10	1.01-1.19	0.027		0.644
		Simple mode	26	1.15	0.93-1.42	0.219		
		Weighted mode	26	1.09	0.96-1.23	0.205		
NKT AC								
	26.70	MR Egger	34	1.09	0.95-1.27	0.234	0.817	0.586
		Weighted median	34	1.05	0.92-1.21	0.456		
		Inverse variance weighted	34	1.11	1.02-1.21	0.017		0.632
		Simple mode	34	1.09	0.87-1.36	0.458		
		Weighted mode	34	1.05	0.91-1.21	0.495		
MFI								
CD8 on CD28- CD8br								
	24.46	MR Egger	20	0.75	0.50-1.14	0.196	0.564	0.623
		Weighted median	20	0.81	0.66-1.00	0.043		
		Inverse variance weighted	20	0.85	0.74-0.97	0.017		0.664
		Simple mode	20	0.82	0.58-1.16	0.273		
		Weighted mode	20	0.83	0.63-1.08	0.185		
CD62L on monocyte								
	29.86	MR Egger	25	0.89	0.83-0.97	0.013	0.599	0.874
		Weighted median	25	0.92	0.83-1.01	0.075		
		Inverse variance weighted	25	0.91	0.85-0.97	0.002		0.894
		Simple mode	25	1.02	0.86-1.21	0.798		
		Weighted mode	25	0.91	0.84-0.98	0.022		

CCR2 on CD14+ CD16- monocyte								
	25.09	MR Egger	25	0.96	0.92-1.01	0.126	0.824	0.451
		Weighted median	25	0.97	0.92-1.02	0.254		
		Inverse variance weighted	25	0.96	0.93-1.00	0.044		0.507
		Simple mode	25	0.93	0.83-1.03	0.161		
		Weighted mode	25	0.97	0.92-1.01	0.156		
CD33 on CD33br HLA DR+ CD14dim								
	66.86	MR Egger	27	1.04	0.97-1.12	0.237	0.827	0.377
		Weighted median	27	1.02	0.96-1.08	0.580		
		Inverse variance weighted	27	1.05	1.00-1.10	0.029		0.429
		Simple mode	27	1.09	0.97-1.22	0.143		
		Weighted mode	27	1.02	0.97-1.09	0.428		
CD39 on CD39+ CD4+								
	144.67	MR Egger	20	1.03	0.93-1.13	0.610	0.314	0.430
		Weighted median	20	1.03	0.96-1.11	0.389		
		Inverse variance weighted	20	1.06	1.00-1.13	0.043		0.425
		Simple mode	20	1.29	1.03-1.61	0.038		
		Weighted mode	20	1.03	0.96-1.11	0.453		
CX3CR1 on CD14- CD16-								
	35.40	MR Egger	19	1.14	1.07-1.22	0.001	0.006	0.632
		Weighted median	19	1.05	0.98-1.13	0.198		
		Inverse variance weighted	19	1.07	1.00-1.13	0.039		0.137
		Simple mode	19	1.04	0.93-1.15	0.532		
		Weighted mode	19	1.07	1.00-1.14	0.061		

CD3 on CD39+ secreting Treg								
	38.62	MR Egger	26	1.18	1.03-1.35	0.028	0.168	0.559
		Weighted median	26	1.14	1.01-1.28	0.033		
		Inverse variance weighted	26	1.09	1.00-1.18	0.044		0.498
		Simple mode	26	1.07	0.90-1.29	0.454		
		Weighted mode	26	1.12	0.99-1.27	0.080		
CD28 on CD39+ secreting Treg								
	36.46	MR Egger	21	1.03	0.92-1.15	0.631	0.156	0.727
		Weighted median	21	1.07	0.96-1.19	0.212		
		Inverse variance weighted	21	1.09	1.01-1.18	0.031		0.645
		Simple mode	21	1.22	1.03-1.45	0.036		
		Weighted mode	21	1.06	0.96-1.17	0.268		
CD28 on CD39+ CD4+								
	62.01	MR Egger	15	1.00	0.88-1.15	0.982	0.152	0.986
		Weighted median	15	1.04	0.94-1.16	0.417		
		Inverse variance weighted	15	1.09	1.01-1.18	0.036		0.944
		Simple mode	15	1.07	0.91-1.25	0.415		
		Weighted mode	15	1.05	0.94-1.16	0.395		
HVEM on CD4+								
	23.86	MR Egger	18	1.11	0.98-1.26	0.133	0.835	0.267
		Weighted median	18	1.07	0.96-1.20	0.210		
		Inverse variance weighted	18	1.10	1.02-1.19	0.017		0.324
		Simple mode	18	1.07	0.91-1.24	0.422		
		Weighted mode	18	1.07	0.96-1.19	0.247		
CD28 on CD45RA- CD4 not Treg								

	37.71	MR Egger	13	1.20	0.94-1.52	0.168	0.744	0.986
		Weighted median	13	1.16	1.00-1.34	0.053		
		Inverse variance weighted	13	1.15	1.03-1.30	0.016		0.992
		Simple mode	13	1.15	0.92-1.44	0.251		
		Weighted mode	13	1.15	0.96-1.37	0.149		
CD25 on activated Treg								
	21.75	MR Egger	17	1.09	0.81-1.47	0.594	0.544	0.597
		Weighted median	17	1.04	0.84-1.29	0.689		
		Inverse variance weighted	17	1.18	1.01-1.37	0.032		0.640
		Simple mode	17	1.03	0.73-1.45	0.868		
		Weighted mode	17	1.04	0.74-1.44	0.840		
Morphological parameter								
SSC-A on HLA DR+ NK								
	36.17	MR Egger	27	0.97	0.89-1.06	0.029	0.235	0.568
		Weighted median	27	1.02	0.93-1.12	0.292		
		Inverse variance weighted	27	0.94	0.88-1.00	0.019		0.539
		Simple mode	27	0.98	0.83-1.15	0.536		
		Weighted mode	27	0.98	0.90-1.08	0.109		
Relative count								
CD4+ CD8dim %leukocyte								
	37.29	MR Egger	13	0.80	0.57-1.13	0.230	0.694	0.668
		Weighted median	13	0.82	0.67-1.01	0.057		
		Inverse variance weighted	13	0.85	0.73-0.99	0.043		0.732
		Simple mode	13	0.81	0.59-1.13	0.242		
		Weighted mode	13	0.81	0.66-1.00	0.073		
HLA DR+ CD8br %T cell								

	32.41	MR Egger	29	1.05	0.97-1.14	0.873	0.419	0.739
		Weighted median	29	1.08	0.99-1.17	0.157		
		Inverse variance weighted	29	1.07	1.01-1.14	0.005		0.751
		Simple mode	29	1.04	0.93-1.17	0.588		
		Weighted mode	29	1.06	0.98-1.14	0.573		
CD39+ resting Treg %resting Treg								
	109.90	MR Egger	27	1.07	0.99-1.16	0.137	0.798	0.673
		Weighted median	27	1.04	0.97-1.12	0.068		
		Inverse variance weighted	27	1.08	1.02-1.14	0.019		0.720
		Simple mode	27	1.08	0.93-1.24	0.441		
		Weighted mode	27	1.07	1.00-1.14	0.113		
NKT %T cell								
	27.82	MR Egger	31	1.12	0.98-1.28	0.107	0.707	0.131
		Weighted median	31	1.04	0.92-1.17	0.557		
		Inverse variance weighted	31	1.10	1.01-1.20	0.037		0.155
		Simple mode	31	1.13	0.91-1.42	0.279		
		Weighted mode	31	1.05	0.93-1.17	0.452		
CD11c+ monocyte %monocyte								
	22.26	MR Egger	20	1.07	0.89-1.29	0.455	0.409	0.772
		Weighted median	20	1.17	1.00-1.38	0.054		
		Inverse variance weighted	20	1.15	1.03-1.29	0.009		0.782
		Simple mode	20	1.24	0.96-1.60	0.116		
		Weighted mode	20	1.19	1.01-1.41	0.050		

Abbreviations: CI, confidence interval; PC, pancreatic cancer; MR, Mendelian randomization; OR, odds ratio; SNP, single nucleotide polymorphism.
